# Increased Nerve Growth Factor Signaling in Sensory Neurons of Early Diabetic Rats Is Corrected by Electroacupuncture

**DOI:** 10.1155/2013/652735

**Published:** 2013-04-21

**Authors:** Stefania Lucia Nori, Maria Luisa Rocco, Fulvio Florenzano, Maria Teresa Ciotti, Luigi Aloe, Luigi Manni

**Affiliations:** ^1^Department of Pharmaceutical and Biomedical Sciences (FARMABIOMED) Nanomates, University of Salerno, Via Ponte don Melillo, 84084 Fisciano, Italy; ^2^Institute of Cellular Biology and Neurobiology, National Research Council (CNR), Via del Fosso di Fiorano 64, 00143 Rome, Italy; ^3^Confocal Microscopy Unit, European Brain Research Institute (EBRI), Institute of Cellular Biology and Neurobiology, National Research Council (CNR), Via del Fosso di Fiorano 64, 00143 Rome, Italy; ^4^Institute of Translational Pharmacology, National Research Council (CNR), Via del Fosso del Cavaliere 100, 00133 Rome, Italy

## Abstract

Diabetic polyneuropathy (DPN), characterized by early hyperalgesia and increased nerve growth factor (NGF), evolves in late irreversible neuropathic symptoms with reduced NGF support to sensory neurons. Electroacupuncture (EA) modulates NGF in the peripheral nervous system, being effective for the treatment of DPN symptoms. We hypothesize that NGF plays an important pathogenic role in DPN development, while EA could be useful in the therapy of DPN by modulating NGF expression/activity. Diabetes was induced in rats by streptozotocin (STZ) injection. One week after STZ, EA was started and continued for three weeks. NGF system and hyperalgesia-related mediators were analyzed in the dorsal root ganglia (DRG) and in their spinal cord and skin innervation territories. Our results show that four weeks long diabetes increased NGF and NGF receptors and deregulated intracellular signaling mediators of DRG neurons hypersensitization; EA in diabetic rats decreased NGF and NGF receptors, normalized c-Jun N-terminal and p38 kinases activation, decreased transient receptor potential vanilloid-1 ion channel, and possibly activated the nuclear factor kappa-light-chain-enhancer of activated B cells (Nf-**κ**B). In conclusion, NGF signaling deregulation might play an important role in the development of DPN. EA represents a supportive tool to control DPN development by modulating NGF signaling in diabetes-targeted neurons.

## 1. Introduction

Sensory polyneuropathy is a major complication of type 1 diabetes, still lacking adequate treatment [1]. As a consequence of metabolic dysfunctions, diabetic polyneuropathy (DPN) develops in an early responsive “metabolic phase” and a late nonresponsive “structural phase” [1]. The early DPN is characterized by peripheral nerve dysfunctions, being reversible by common euglycemic therapies [1] and presenting some peculiar features that could account for the pathogenesis of late DPN. The early neuropathic symptoms develop as thermal hyperalgesia [2–5] associated with increased neurotrophism [3]. The late DPN presents irreversible neuropathic symptoms, such as thermal hypoalgesia and mechanical allodynia, associated with a reduction in neurotrophic support to peripheral nervous system (PNS) neurons and with neuronal sufferance and atrophy [1].

Nerve growth factor (NGF) is essential for the development and functional maintenance of dorsal root ganglion (DRG) sensory neurons, which are primarily targeted by diabetes [6], and has been indicated as a possible therapeutic for peripheral neuropathies [7]. Components of the NGF downstream signaling system—namely, the extracellular signal-regulated kinases 1/2 (ERK1/2), the c-jun N-terminal kinase (JNK), and the Akt and the p38 kinases—activated after the challenge of the NGF receptor tyrosine kinase A (TrkA) and/or the p75 pan-neurotrophin receptor (p75^NTR^) were found deregulated in experimental diabetes [8, 9]. NGF supply in animal models of diabetic neuropathies reverses neuropathic signs by normalizing the PNS activity [10, 11]. Clinical trials have been performed on diabetic patients [7], evidencing positive outcomes but also important side effects, correlated to the action of NGF on the pain system, limiting the development of NGF-based therapies for DPN [7]. Thus, therapies based on the modulation of the endogenous NGF system gained consensus [12, 13], with the purpose of avoiding undesired side effects.

Traditional Chinese acupuncture and its western derivate electroacupuncture (EA) have been proven effective in the therapy of neuropathic pain [14, 15], and their neurophysiologic correlates are actually subjects of extensive investigation [15–17]. We previously demonstrated that EA could modulate NGF system in animal models of neuronal and neuroendocrine diseases [3, 16]. Moreover, EA was effective in the treatment of neuropathic pain in diabetes [3, 14]. Our working hypothesis, therefore, is that the possible action of EA on diabetic neuropathy could be mediated through the NGF signaling pathway.

Here, we investigated the possible regulation of the NGF system in primary sensory neurons and in their innervation targets, in the early phase of experimental diabetes after EA treatment. We also studied the variation of NGF intracellular mediators since their reported involvement in the development of neuropathic symptoms in the STZ diabetes model [4, 9, 18] as well as the transient receptor potential cation channel subfamily V member 1 (TRPV1) due to its known connection with both the NGF system [19] and DPN [18].

## 2. Materials and Methods

### 2.1. Animals

Adult female Sprague-Dawley rats (Harlan-Nossan, Correzzana, Italy) weighing 200–220 g were housed under constant environmental conditions. Animals care and experiments were conducted in conformity with Italian and international laws and policies (EEC Council Directive 86/609, OJ L 358,1,12 December 1987; NIH Guide for the Care and Use of Laboratory Animals, NIH Publication no. 85-23, 1985). The experimental protocol (no. 10/2011) was approved by the Italian Ministry of Health. All surgeries were performed under chloral hydrate anesthesia, and all efforts were made to reduce the number of animals used and to minimize their sufferance.

### 2.2. Experimental Plan and Tissue Collection

Rats were treated with a single i.p. injection of 65** **mg/kg streptozotocin (STZ: S0130, Sigma-Aldrich S.r.l., St. Louis, MO, USA) dissolved in citrate buffer pH 4.5 to induce type 1 diabetes [20]. One week after STZ, glycemia was checked by blood glucose analyzer (Accutrend GC, Roche Diagnostic GmbH, Germany). Rats with blood glucose levels above 300 mg/dL were enrolled in the STZ experimental groups. Thirty-six rats were divided as follows: untreated rats (control, *n* = 12) were injected once i.p. with 20** **mM citrate, pH 4.5; STZ-injected (STZ, *n* = 12) STZ-injected, and electroacupuncture-treated rats (STZ + EA, *n* = 12) were treated twice a week with low-frequency EA for 3 consecutive weeks starting 1 week after diabetes induction. Control of the EA treatment trough minimal or superficial sensory stimulation (i.e., sham-EA) in diabetic rats was omitted, since it has been demonstrated that it does not represent an inert intervention, often arising significant responses based on the same anatomic and physiological substrates activated by acupuncture itself [21]. The treatment with EA in healthy rats was also omitted because of the well-demonstrated different effects of EA treatment in healthy and diseased subjects [21].

Four weeks after STZ and one day after the last EA session, 10 rats in each group were killed by decapitation, serum, hindpaw skin, lumbar (L3–L5) DRG, and lumbar spinal cord, collected and quickly frozen and stored at −80°C until processed for assays. Two rats for each group were anesthetized with 400 mg/kg of chloral hydrate and transcardially perfused with 4% paraformaldehyde in PBS. Samples of the glabrous hindpaw skin (2 mm diameter, 1 mm thick circle-shaped specimens collected by a disposable biopsy punch from the sole of the hindpaw) and of the lumbar spinal cord tissues were removed and postfixed for 24 hours. Then, tissue samples were transferred in 30% sucrose/PBS solution and sectioned at a sliding freezing microtome (Leica, Wetzlar, Germany). Forty-micrometer-thick sections were collected in 0.05% sodium azide/PBS and stored at 4°C until used.

### 2.3. Electroacupuncture

Rats from the EA group were exposed to 30 minutes of EA stimulation twice a week for 3 weeks [22]. During each treatment, rats were sedated with an i.p. injection of chloral hydrate (200 mg/kg). Stimulations were applied bilaterally at the traditional acupoint ST36 (on the anterior lateral side of the leg close to the anterior crest of the tibia). Such stimulation has been previously proven effective for the modulation of endogenous NGF in diabetic peripheral neuropathy [3]. The needles (Hegu AB, Sweden) were inserted at the same point bilaterally to depths of 0.3–0.5 cm and attached bilaterally via clips electrodes to an electrical stimulator (ACUS II, Cefar, Sweden). Stimulation was a low burst frequency of 2 Hz; each pulse was a square electric wave with a duration of 180 *μ*sec, a length of 0.1 sec, and internal burst frequency of 80 Hz. The intensity (1.0–1.5 mA) was monitored by checking for local muscle contractions to reflect the activation of muscle-nerve afferents. Control and STZ rats were manipulated and sedated in a manner similar to the STZ + EA rats.

### 2.4. Plasma Glucose and Insulin

Glucose content in plasma derived from blood collected when animals were sacrificed was checked by a commercially available blood glucose analyzer (Accutrend GC, Roche Diagnostic GmbH, Germany). Insulin levels were assessed by Insulin Rat ELISA test (S-1238) from Bachem AG (Bubendorf, Switzerland), following manufacturer's instructions.

### 2.5. NGF Assay

DRGs were homogenized as previously described [3], and protein concentrations were measured by the Biorad DC Protein assay (Biorad Labs, Hercules, CA, USA). NGF in DRG extracts (*n* = 10 for each experimental group) was measured by commercial ELISA (DY556, R&D Systems, MN, USA), following manufacturer's instructions.

### 2.6. Western Blot

DRGs extracts were also used for Western blots of NGF receptors. TrkA and p75^NTR^, ERK1-2, Akt, JNK, p38, phospho-I*κ*B-*α*, phospho-NF-*κ*B-p65, TRPV-1, and GAPDH protein tissue contents (see Table 1 for a detailed list of antibodies used). Samples (20 *μ*g of total protein) were diluted with loading buffer [3], separated by SDS-PAGE, and electrophoretically transferred to polyvinylidene fluoride (PVDF) membrane. The membranes were incubated for 1 hour at room temperature with 5% of nonfat dry milk dissolved in 10 mmol/L Tris, pH 7.5, 100 mmol/L NaCl, and 0.1% Tween-20 (TBST). Membranes were washed in TBST and incubated overnight at 4°C with primary antibodies (Table 1). Membranes were then washed in TBST and incubated for 1 hour with either horseradish peroxidase-conjugated anti-rabbit IgG (Cat. Nr. 7074) or horseradish peroxidase-conjugated anti-mouse IgG (Cat. Nr. 7076) from Cell Signaling Technology (Danvers, MA, USA) as the secondary antibody. The blots were developed with ECL-HRP substrate (Merck Millipore, Darmstadt, Germany) as the chromophore. The public domain ImageJ software (http://rsb.info.nih.gov/ij/) was used for gel densitometry and protein quantification following the method described in [23]. The GAPDH was used as a normalizing factor. Statistical evaluation was performed on 3 separate gels run/blots carried out using 3 different sets of samples (*n* = 9 for each experimental group).

### 2.7. RNA Isolation and cDNA Synthesis

Total RNA was extracted from DRG, paw skin, and spinal cord samples by SV Total RNA Isolation Kit (Promega Italia, Milan, Italy), following manufacturer's instructions. DNase I (included in the kit) digestion was performed to eliminate DNA contamination. RNA concentration was determined by NanoDrop ND-1000 (NanoDrop Technologies, Wilmington, DE, USA). First-strand cDNA was synthesized from 0.5 *μ*g total RNA using the GoScript Reverse Transcription System (Promega Italia, Milan, Italy) following manufacturer's instructions.

### 2.8. Real-Time PCR

Gene expression of NGF in the spinal cord and hindpaw skin and of TrkA and p75^NTR^ in the DRGs were analyzed by real-time PCR using inventoried TaqMan assays from Applied Biosystems (Life Technologies Corp., CA, USA). The assays codes were Rn01533872_m1 (NGF), Rn00561634_m1 (p75^NTR^), and RN00572130_m1 (TrkA). GAPDH (4352338E, Applied Biosystems) was used as an endogenous control to allow for relative gene expression quantification. Thermal cycling and fluorescence detection were performed with an ABI Prism 7900HT Sequence Detection System with SDS Software 2.1 (Applied Biosystems). Thermal cycling conditions were 2 min at 50°C and 10 min at 94.5°C, followed by 40 cycles of 15 s at 95°C and 1 min at 60°C. Relative gene expression was calculated using the 2^−ΔΔCt^ method [24].

### 2.9. Confocal Immunofluorescence

TrkA and p75^NTR^ distribution in the hind paw skin and spinal cord were analyzed by immunofluorescence. Forty-micrometer-thick tissue sections were preincubated with 10% of donkey serum in PBS containing 0.1% Triton X-100 (PBST) for 2 hours and then incubated overnight at 4°C with primary antibodies against TrkA (1 : 100) and p75^NTR^ (1 : 200) listed in Table 1. To assess for staining specificity, the first antibody was replaced by purified nonspecific rabbit or mouse IgG. After washing with PBST, the sections were incubated for 1 hour at room temperature with Alexa Fluor 488 donkey anti-rabbit IgG (1 : 200) and Alexa Fluor 555 donkey anti-mouse IgG (1 : 200). After two rinses in PBS and 10 min incubation with the DAPI solution (Molecular Probes, Invitrogen, Italy) for nuclei visualization, sections were coverslipped and examined under a confocal laser scanning microscope (Leica SP5, Leica Microsystems, Germany) under sequential mode to avoid crosstalk between channels. The confocal image acquisition was conducted so that all samples were imaged using consistent settings for laser power and detector gain. Boundaries and subdivisions of the spinal cord structures were identified with reference to the Paxinos atlas [25]. Image processing and final figures were done by using Adobe Photoshop 7 and Adobe Illustrator 10.

### 2.10. Image Analysis

Image analysis of p75^NTR^ and TrkA double immunofluorescence was performed by Imaris 7.4 (Bitplane A.G.) software on five different images derived from each experimental group. For the skin, a mask was manually drawn on the basal cell layer and for the spinal cord on the laminae I to III. All the parameters were selectively evaluated only on these histological structure. Morphological parameters under analysis were tissue intensity, vesicles/terminals intensity, vesicles/terminals number, diameter, and colocalization which were evaluated by using surface, spots, and Coloc Imaris modules. To determine fluorescent signal colocalization between different channels, a median filter was applied to reduce the background noise, and the degree of overlap was characterized by Manders' and Pearson's coefficient.

### 2.11. Statistic

Data were analyzed by the GraphPad 5 software (GraphPad Software Inc., USA). Western blot (*n* = 9 for each experimental group), ELISA and real-time PCR (*n* = 10 for each experimental group), and image analysis (*n* = 5 for each experimental group) data were then evaluated by one-way ANOVA and data expressed as mean ± SD. Post hoc comparisons within different experimental groups were performed using Tukey's HSD test. A *P* value less than 0.05 was considered significant. 

## 3. Results

### 3.1. Blood Glucose and Insulin after STZ and EA

STZ treatment induced a significant increase of plasma glucose, (STZ: 621.0 ± 42.4 mg/dL; controls: 94.4 ± 12.3 mg/dL, *P* < 0.05) that was concomitant with a significant decrease of plasma insulin (STZ: 1.14 ± 1.05 pg/mL; Controls: 2.88 ± 1.18 pg/mL, *P* < 0.05). EA induced a slight, nonsignificant improvement in plasma glucose (STZ + EA: 575.4 ± 95.9 mg/dL; *P* > 0.05 when STZ and STZ + EA groups were compared) and did not affect plasma insulin (STZ + EA: 1.48 ± 1.14 pg/mL; *P* > 0.05 when STZ and STZ + EA groups were compared).

### 3.2. NGF Protein Was Increased following STZ and Normalized by EA

**Figure 1:**
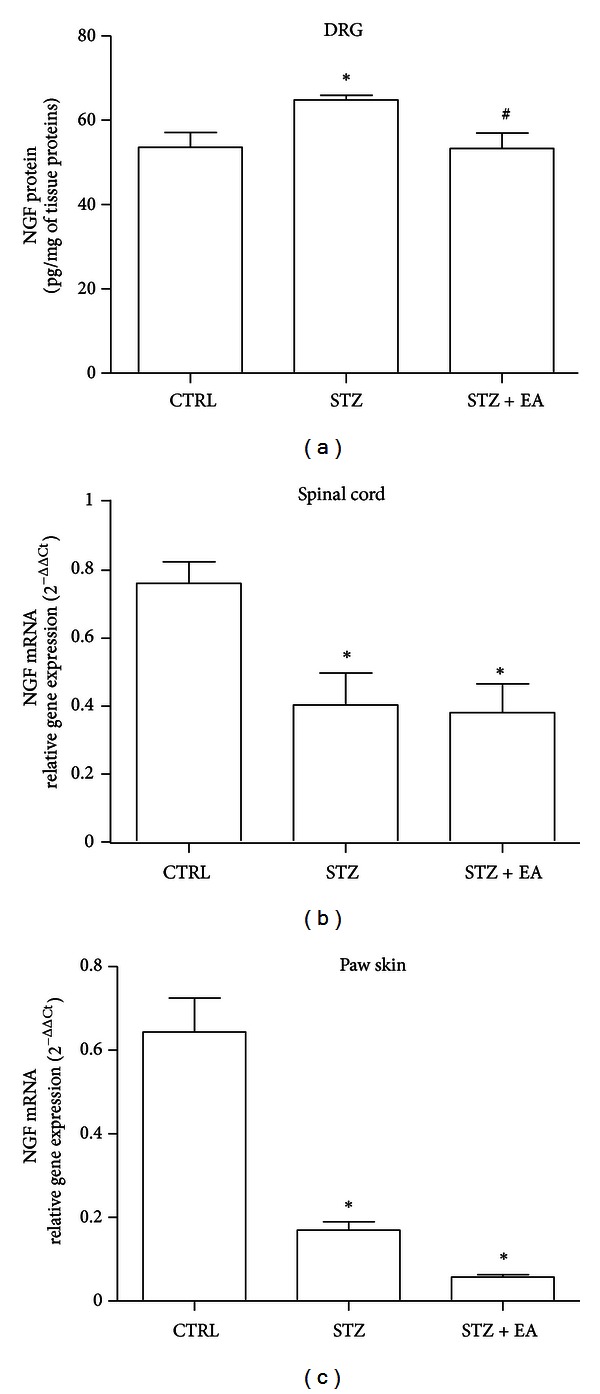
NGF protein and mRNA levels following STZ and EA. Four weeks after STZ injection, an elevation of NGF protein content (a), as measured by ELISA, was found in the lumbar DRGs of adult rats. EA applied for three weeks, starting one week after STZ, restored baseline levels of DRG's NGF in diabetic rats. Data from NGF ELISA are presented as mean ± SD; *n* = 10 for each experimental group. *P < 0.05 versus control group. ^#^ *P* < 0.05 versus STZ group. NGF mRNA was measured by semiquantitative real-time PCR at anatomical sites responsible for the production of NGF supplied to DRG neurons. The lumbar spinal cord (b) and hindpaw skin (c) NGF mRNA were found decreased four weeks after diabetes induction, and the three-week treatment with EA did not affecte STZ-induced NGF mRNA decrease. Relative NGF gene expression values were calculated using the 2^−ΔΔCt^ method and the GAPDH used for normalization. Data are expressed as mean 2^−ΔΔCt^  ± SD; *n* = 10 for each experimental group. *P < 0.05 versus Control group.

STZ caused a significant +33% increase of NGF protein content in the DRGs (Figure 1), that was corrected by EA. To ascertain the causes of this modification, we investigated NGF mRNA production in the DRG innervations territories, that are the production sites for the NGF used by DRG neurons. STZ provoked a marked reduction in NGF mRNA (Figure 1) in both the hind paw skin (STZ versus control group: −73%, *P* < 0.05) and spinal cord (STZ versus control group: −48%, *P* < 0.05). EA did not correct such reductions, suggesting a possible control of the EA on the NGF protein production or uptake/transport rather than on the NGF gene expression.

### 3.3. NGF Receptor Levels Are Modified following STZ Induction and EA Treatment

**Figure 2:**
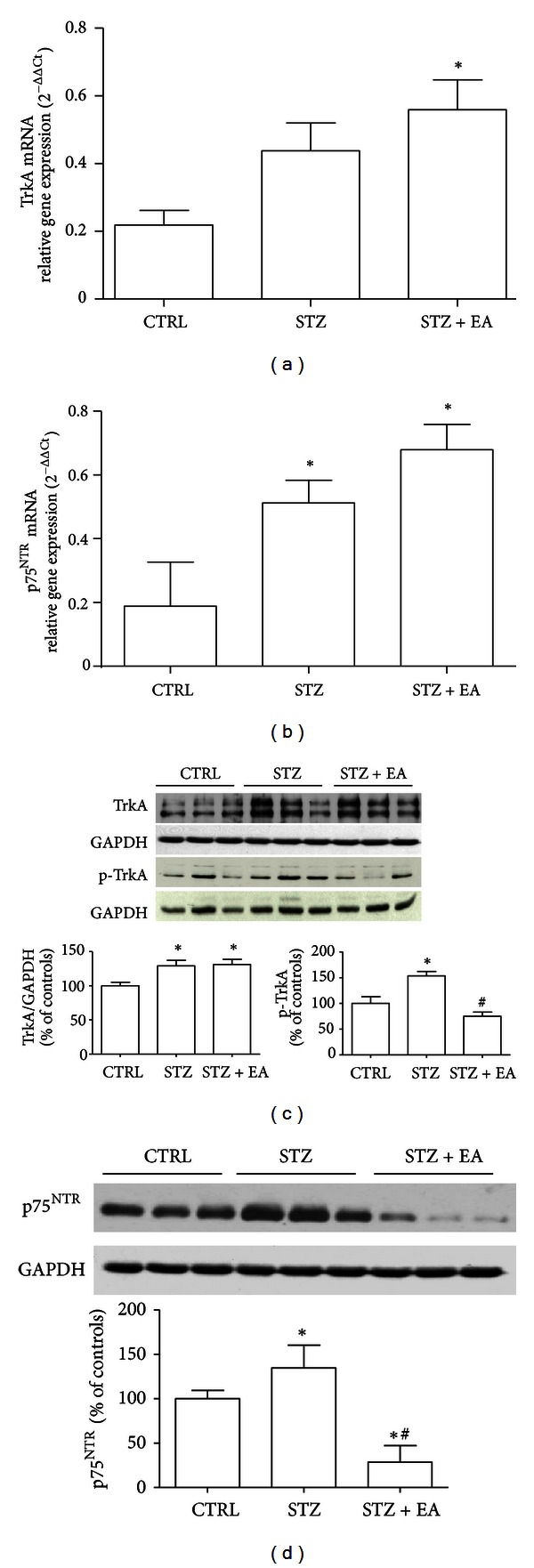
Effects of electroacupuncture on NGF receptors TrkA and p75^NTR^ in the DRGs of diabetic rats. Relative TrkA (a) and p75^NTR^ (b) gene expression was analyzed in DRGs by real-time PCR and data obtained using the 2^−ΔΔCt^ method. STZ induced a nonsignificant increase of TrkA mRNA in the DRGs (a) that reached a significant value after EA. STZ induced a significant increase of p75^NTR^ mRNA in the DRGs (b), while EA did not affect such increase. Data presented in (a) and (b) are expressed as mean 2^−ΔΔCt^  ± SD; *n* = 10 for each experimental group; *P < 0.05 versus control group. Representative TrkA and p_Tyr496_-TrkA (p-TrkA) Western blots of three samples for each experimental group are presented (c). Densitometry of three separate gels run/blots (c) revealed that four weeks after diabetes induction, both the high-affinity NGF receptor TrkA and its activated form, the p_Tyr496_-TrkA, in DRGs were increased. EA in diabetic animals induced a significant deactivation of the TrkA receptor, as indicated by the significant decrease of the phospho-TrkA in the STZ + EA group. Representative p75^NTR^ Western blot of three samples for each experimental group is also presented (d). Densitometry of three separate gels run/blots (d) revealed that STZ induced a significant increase of the p75^NTR^ in DRGs. EA reduced p75^NTR^ protein content in diabetic DRGs well below control level. Data presented in (c) and (d) are obtained after normalization with GAPDH bands. Western blots data (*n* = 9) are all expressed as % of controls mean ± SD; *P < 0.05 versus Control group. ^#^ *P* < 0.05 versus STZ group.

TrkA mRNA in DRGs (Figure 2(a)) showed a tendency to increase through the experimental groups that became significant after EA (STZ + EA versus control group: +156%, *P* < 0.05). Western blots densitometry (Figure 2(c)) revealed that the TrkA protein in the DRGs of diabetic animals underwent a significant increase (STZ versus controls: +29%, *P* < 0.05; data not shown), while EA did not exert significant effects on TrkA protein content (STZ + EA versus STZ: +2%, *P* > 0.05). The p_Tyr496_-TrkA, the activated form of TrkA, was significantly increased by diabetes (STZ versus controls: +54%, *P* < 0.05), indicating an augmented NGF signaling. EA induced a significant reduction of p_Tyr496_-TrkA in diabetic animals (STZ + EA versus STZ: −24.7%, *P* < 0.05), indicating an inhibitory effect of EA upon TrkA activation.

We also investigated the modifications of the p75^NTR^ receptor, known to be activated by NGF in neuropathic conditions [26]. The mRNA-p75^NTR^ (Figure 2(b)) was significantly increased in DRGs of both diabetic and EA rats. Western blot (Figure 2(d)) revealed that p75^NTR^ protein was increased (+35%, *P* < 0.05) above controls by STZ, while EA decreased p75^NTR^ content in the DRGs well below control level (STZ + EA versus STZ: −71.5%, *P* < 0.05), indicating a parallel effect of STZ and EA on the p75^NTR^ and its ligand NGF (see Figure 1), at least at protein level.

### 3.4. TrkA/p75^NTR^ Immunofluorescence in Hind Paw Epidermis

**Figure 3:**
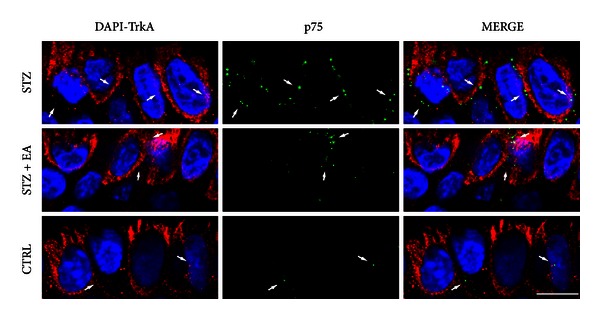
Confocal microscopy of TrkA and p75^NTR^ double immunofluorescence in the basal epidermal layer. To allow better p75^NTR^ vesicles visualization, the red signal was changed to green by colour palette modification. First column DAPI/TrKA (blue, red palette assigned), second column p75^NTR^ (green palette assigned), third column DAPI/TrKA/p75 (MERGE). The amount of p75^NTR^ cytoplasmatic immunoreactive vesicles increases in the basal epidermal layer of the STZ group (first row, arrows) on very low fluorescence background. After EA treatment and the p75^NTR^ cytoplasmatic immunoreactive vesicles were decreased (second row, arrows), while, in the basal epidermal layer of the control group p75^NTR^ only few immunoreactive vesicles were detected (third row, arrows). TrkA did not show variations in the three experimental groups. TrkA immunoreactivity is characterized by a medium-high tissue fluorescence background decorated by many immunoreactive vesicles which often were confluent. Scale bar: 8 *μ*m.

Confocal microscopy analysis of TrkA and p75^NTR^ double immunofluorescence in the skin (Figure 3 and Supplementary Figure 1; see Supplementary Material available online at http://doi.org.10.1155/2013/652735) revealed a selective increase of p75^NTR^ immunoreactivity in the epidermal basal cell layer after STZ which was partially reverted by EA (Figure 3, Supplementary Figure 1 and Tables 2 and 3). The increased p75^NTR^ expression in the STZ group, compared to controls, was evident as tissue fluorescence intensity (+157%), as vesicles intensity (+401%), and as the number of immunopositive vesicles (+206%) in the cellular cytoplasm (Table 2). EA treatment was able to significantly reverse only the modification in the number of immunopositive vesicles (−56%).

Spatial distribution analyses of TrkA and p75^NTR^ immunofluorescence signals showed the substantial lack of colocalization in all three experimental groups (Figure 3, Supplementary Figure 1 and Table 2). The slight increase above the 0.5 value in the Manders' coefficient of TrkA versus p75^NTR^ in all three experimental groups (Table 2) suggests that in the skin TrkA immunoreactivity is present in p75^NTR^-positive structures and could participate to p75^NTR^ signaling.

### 3.5. TrkA/p75^NTR^ Immunofluorescence in the Spinal Cord

**Figure 4:**
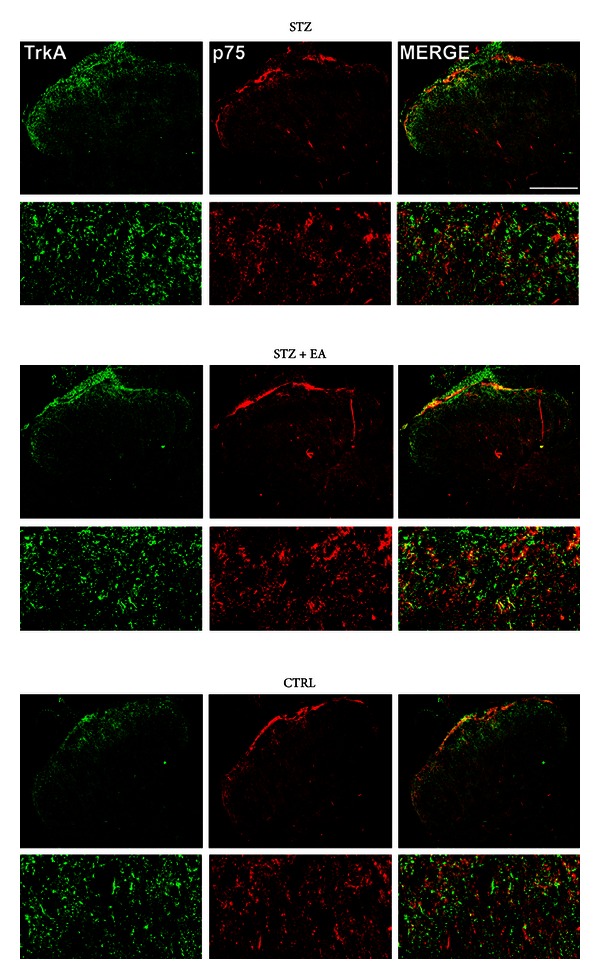
Low and high magnification confocal microscopy images of TrkA and p75^NTR^ double immunofluorescence in the spinal cord of STZ, STZ + EA, and Control groups. In all three groups, TrkA and p75^NTR^ immunoreactivity appeared mainly expressed in the superficial dorsal horn and surrounding white matter as terminals and small fibers. An intense p75^NTR^ immunoreactivity was observed in bundles of fibers running tangentially to the superficial dorsal horn in the white matter. In the same region, a medium intensity TrkA immunoreactivity was present in wisps of small fibers running radially to the superficial dorsal horn. In the laminae I and II of the dorsal horn, both TrkA and p75^NTR^ immunoreactivity appeared as positive terminals or small fibers which very often showed juxtaposition relations but not colocalization. Scale bar: 15 *μ*m.

Confocal microscopy analysis of TrkA and p75^NTR^ double immunofluorescence in the superficial laminae of the dorsal horn (Figure 4) revealed a significant increase of TrkA immunoreactivity after STZ which was reverted by EA (Figure 4 and Table 3). At variance, p75^NTR^ immunoreactivity after STZ significantly decreased, and EA treatment was not able to reverse this modification (Figure 4 and Table 3). In all three experimental groups, both TrkA and p75^NTR^ appeared confined to terminals and to some small fibers mainly distributed in laminae I to III of the dorsal horn (Figure 4). Spatial distribution analyses of immunofluorescence signals showed the lack of a significant colocalization in all three experimental groups (Table 2).

In the STZ group, the TrkA tissue and terminals fluorescence intensity showed a significant increase (+255% and +31%, resp.) compared to the controls (Figure 4 and Tables 2 and 3). At variance, p75^NTR^ significantly decreased (−43%) only the terminals fluorescence intensity (Figure 4 and Tables 2 and 3). In the STZ + EA group, the TrkA tissue fluorescence intensity showed a significant (−50%) decrease when compared to the STZ group, while p75^NTR^ did not show significant variations (Figure 4 and Tables 2 and 3).

### 3.6. Modifications of NGF Signaling in DRGs following STZ Induction and EA

**Figure 5:**
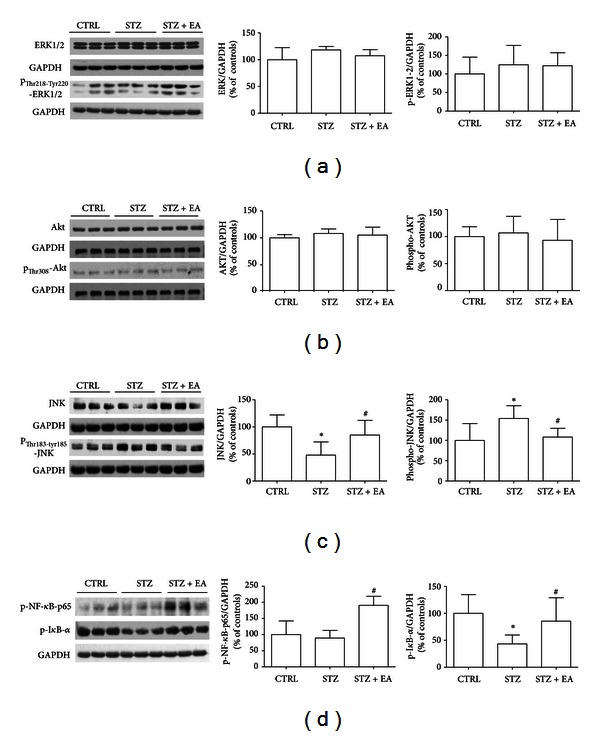
Variations of downstream NGF signaling after STZ and EA in DRGs. Representative Western blot of three samples for each experimental group is presented in each panel, together with data from densitometry analysis of three separate gel/blot runs (*n* = 9). The analysis of the total ERK1/2 and phospho_Thr218-Tyr220_-ERK1/2 (p-ERK1/2) (a) and the total Akt and phospho_Thr308_-Akt (p-Akt) (b) revealed that any significant variation in expression and the activation of these two TrkA-related downstream signaling kinases was induced by experimental treatments in the DRGs. The JNK that is known to be part of the p75^NTR^ downstream signaling machinery (c) was decreased four weeks after STZ, while phospho_Thr183-Tyr185_-JNK (p-JNK) was increased. EA normalized both variations. The presence of the p75^NTR^ downstream signaling molecule and phosphorylated NF-*κ*B-p65 complex—representing an index of the nuclear translocation activity of the transcription factor NF-*κ*B—was unaffected by STZ (d), while EA greatly enhanced phospho-NF-*κ*B-p65 presence in DRGs of diabetic rats, suggesting an augmented activity of the factor. Coherently, STZ lowered the phosphorylation of the I*κ*B-*α* below controls level, suggesting an increase of its repressive activity upon NF-*κ*B (d); EA in diabetic rats counteracted such an effect, significantly improving I*κ*B-*α* phosphorylation versus STZ group, further indicating a decreased repression of NF-*κ*B activity induced by EA. Data presented in ((a)–(d)) are percent variations from the mean of control group, obtained after normalization with GAPDH band integrated optical density. Data are expressed as % of the mean of controls (*n* = 9) ± SD *P < 0.05 versus control group. ^#^ *P* < 0.05 versus STZ group.

TrKA activation exerts a downstream effect on the ERK1/2 and Akt kinases, and an activation of kinases involved in the TrkA signaling has been demonstrated in the DRGs of STZ-treated rats [9, 27]. Western blot showed that, in our experimental conditions, no significant variation was induced by STZ or EA in the total and phosphorylated ERK1/2 (Figure 5(a)) and Akt (Figure 5(b)).

For the p75^NTR^ signaling pathways, we analyzed the activation of JNK, known to be proapoptotic and to have hypersensitizing effect in neuropathic models [26]. We found a significant decrease of total JNK and a parallel activation (phosphorylation) of the JNK after STZ (Figure 5(c)). EA reverted to control level both total and phosphorylated forms of the kinase (Figure 5(c)).

In addition, we analyzed the activation of the transcription factor Nf-*κ*B, that is known to be induced by the challenge of p75^NTR^ by NGF, with antiapoptotic and hyposensitizing effects in neuropathic pain [26, 28]. The Nf-*κ*B is maintained into inactive state by the link to its repressor I*κ*B-*α*, that following phosphorylation undergoes degradation with subsequent phosphorylation and nuclear translocation of the Nf-*κ*B [29]. STZ induced a significant decrease of the phospho-I*κ*B-*α* (Figure 5(d). STZ versus controls: −56.5%, *P* < 0.05), suggesting a diabetes-induced repression in the activity of the Nf-*κ*B. EA in diabetic animals induced an increased phosphorylation of the complex Nf-*κ*B-p65 (STZ + EA versus STZ: +91%,  *P* < 0.05) and a concomitant increase in the phosphorylation of the I*κ*B-*α* (STZ + EA versus STZ: +42.1%, *P* < 0.05), suggesting an EA-induced inactivation of the I*κ*B-*α* with a parallel increase in the activity of the Nf-*κ*B.

### 3.7. Modifications of p38 Activation and TRPV1 Content in DRGs following STZ Induction and EA

**Figure 6:**
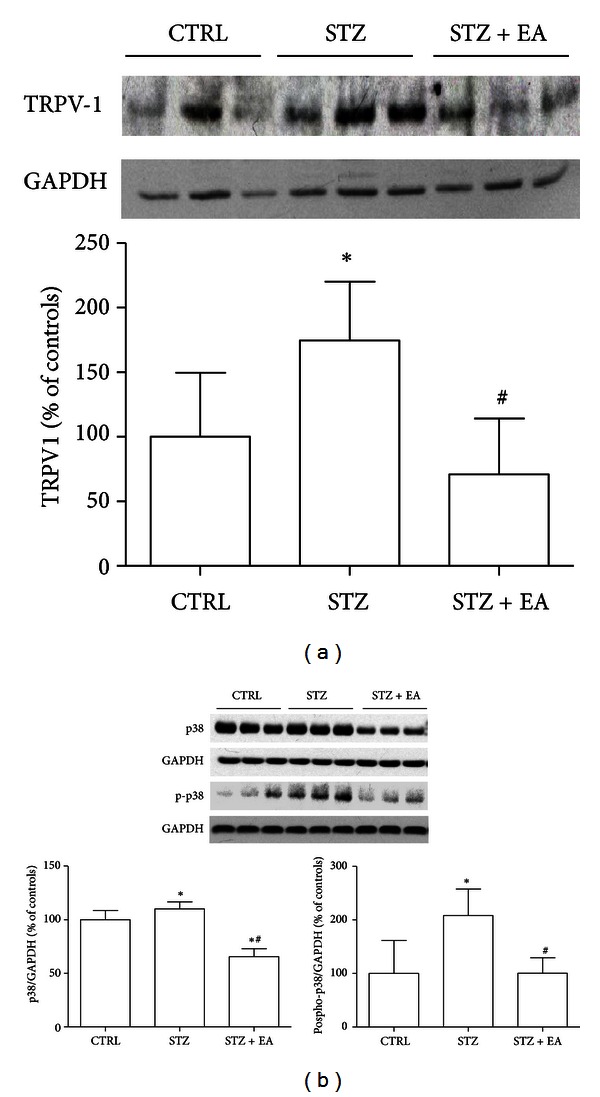
Effects of STZ on p38 kinase and the ion channel TRPV1 are counteracted by EA. Representative Western blot of three samples for each experimental group is presented in each panel, together with data from a densitometry analysis of three separate gel/blot runs (*n* = 9). As revealed by Western blot densitometry, the TRPV1 (a) underwent significant increase, compared to controls in rat DRGs four weeks after diabetes induction. EA normalizes this effect, restoring basal TRPV1 levels. STZ also induced a significant increase in the total kinase p38 and in the phospho_Thr180-Tyr182_-p38 (b). EA in diabetic animals was able to counteract the STZ effects on p38 expression activation, restoring basal levels of both total and phosphorylated p38. Data presented in panel (a) and (b) are obtained after normalization with GAPDH band integrated optical density. Data are expressed as % of controls mean (*n* = 9) ± SD. *P < 0.05 versus control group. ^#^ *P* < 0.05 versus STZ group.

NGF-induced hyperalgesia could be concomitant to the activation of the MAPK p38 that in turn provokes the overexpression of the ion channel TRPV1 in DRG neurons [8, 30]. We investigated the effects of STZ and EA on these well-known intracellular mediators of neuropathic pain. STZ induced a 75% increase in TRPV1 (STZ versus controls, *P* < 0.05) in DRGs (Figure 6(a)) that was concomitant with a slight increase in p38 (STZ versus controls: +10%, *P* < 0.05) and a more substantial increase of phosphorylated p38 (STZ versus controls: +108%, *P* < 0.05; Figure 5(b)). EA in diabetic animals decreased the TRPV1 (STZ + EA versus STZ: −100%, *P* < 0.05), the p38 (STZ + EA versus STZ, −44.5%,  *P* > 0.05), and the phospho-p38 (STZ + EA versus STZ, −108%,  *P* > 0.05), suggesting a normalizing action of the therapy on the p38 activation and in the expression of the pain-mediator TRPV1.

## 4. Discussion

**Figure 7:**
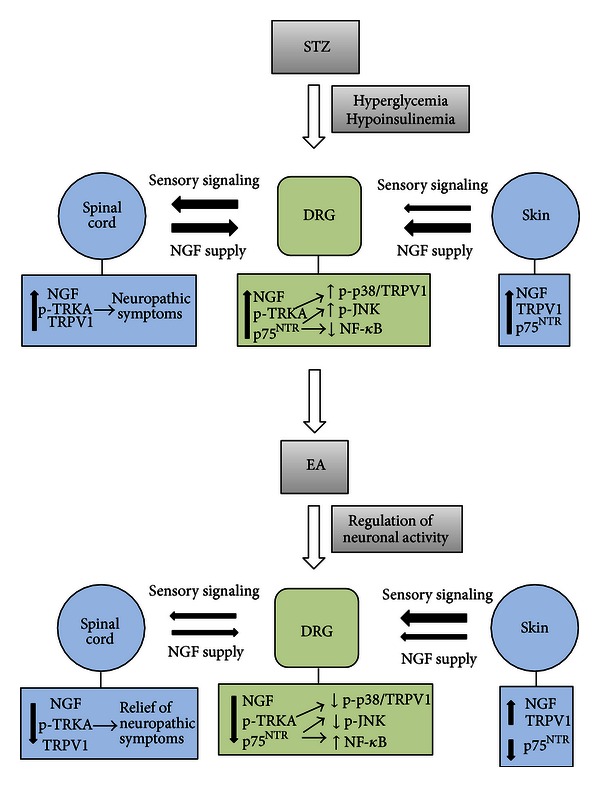
Schematic representation of NGF system activation following STZ and EA in the sensory circuitry linking a peripheral organ (the skin), the sensory neurons located in DRG and their central innervation structure (the spinal cord). The represented mechanism is drawn on the basis of experimental results shown in the present and our previous [3] papers. The DRG neurons receive NGF as a trophic support from both innervation fields. NGF increases throughout the entire circuitry in the first 4 weeks after STZ injection. This NGF increased availability corresponds to the NGF overactivity mediated by TrkA receptor in the spinal cord and by p75^NTR^ in the skin. As a result, both NGF and NGF receptors are overexpressed in the DRG neurons, with a prevalent activation of NGF receptor signaling known to be apoptosis-related and by enhanced presence and possibly activity of the NGF-regulated ion channel TRPV1, known to trigger hyperalgesia as well as neuronal sufferance (see Section 4). Electroacupuncture regulates the activity-dependent NGF synthesis and functions, restores basal nociception and decreases NGF content in the spinal cord and DRG, normalizes TrkA activation in the spinal cord as well as p75^NTR^ presence in the skin and counteracts the STZ-induced events, triggered by JNK/p38 and TRPV1 (possibly a commitment of sensory neurons to apoptosis and neuronal sufferance due to calcium overload, resp.).

Our data demonstrate that in the primary sensory neuron circuitry: (1) early experimental diabetes is characterized by molecular modifications of the NGF signaling system; (2) EA treatment is able to revert the majority of such modifications (Figure 7). The early (4 weeks long) experimental diabetes is characterized by an increased NGF availability for the DRGs sensory neurons, concomitant with increased protein contents of NGF receptors TrkA and p75^NTR^, and by increased TrkA phosphorylation. These diabetes-induced NGF modifications are associated with the activation of JNK and p38, with the repression of the transcription factor Nf-*κ*B and with the upregulation of the ion channel TRPV1. Of particular interest, our data demonstrate that three weeks treatment with low-frequency EA counteracts deregulations of both NGF and NGF receptors, normalizing STZ-induced JNK and p38 activation, decreasing TRPV1, and possibly activating the Nf-*κ*B transcription factor (Figure 7).

As suggested by our previous [3] and present works, where we analyzed NGF responses in early DPN, it is conceivable that NGF plays a main pathogenic role in the early scenario of DPN. In the previous paper [3] we showed that primary sensory neurons, at least in the early development of DPN, have access to larger than normal quantities of NGF, that could originate from their peripheral target, the paw skin [31], or from the central innervation territory, the spinal cord, as demonstrated in spinal injury models [32–34]. In addition, in agreement with data from other laboratories, we have demonstrated early diabetic thermal hyperalgesia [2, 3], that had been coupled not only with increased spinal and skin NGF but also with clues of increased NGF receptors signaling [3]. Support to this hypothesis comes from the presently observed increased TrkA phosphorylation and p75^NTR^ presence, increased MAPKs activity, the possible repression of Nf-*κ*B, and upregulation of the TRPV1. As a limit of our study, it should be stated that these data may represent a mere correlative picture of the NGF system activation after STZ. Indeed, further studies are necessary to clarify: (i) if the effects of STZ and EA on MAPKs and TRPV1 are only mediated by NGF signaling modulation or if other signaling systems are involved; (ii) if neurobehavioural features other than thermal hyperalgesia known to be altered in late diabetic neuropathy and affected by NGF—that is, decreased motor and sensory nerve conduction velocity [35] or increased mechanical sensitivity [36]—would have been affected in our experimental model. However, both activation of JNK and repression of Nf-*κ*B have been linked to p75^NTR^-mediated hyperalgesia [26], and the overexpression of TRPV1 in DRGs after NGF-mediated activation of p38 has been indicated as a cause of inflammation-induced neuropathy and as a possible cause of neuronal distress provoked by the intracellular Ca^2+^ overload mediated by TRPV1 [8, 30]. Activation of JNK and p38 has also been described in animal and human DPN [9]. The emerging picture point at the hyperactivation of NGF signaling system as a main player in the development of DPN and, possibly, in its progress toward the late structural, irreversible neuropathy [1]. In this scenario, p75^NTR^ activation, the possible consequent JNK activation, and Nf-*κ*B repression could play a major role since its well-known role in neurodegenerative diseases [37]. Indeed, our data suggest that TrkA could not actively participate in the early establishment of DPN, at least through the activation of ERK and/or Akt. The possible TrkA-mediated indirect activation of p38; however, should be more specifically addressed.

Data from confocal microscopy give clues about the dynamics of NGF signaling in primary sensory circuitry during the development of experimental DPN. In the skin, the selective p75^NTR^ upregulation following STZ indicates that NGF signaling was shifted from a TrkA/p75^NTR^ physiological state to a p75^NTR^/TRkA “prevalent” state. p75^NTR^ is widely thought to mediate cell death and degenerative phenomena when its activation is concomitant to a high p75^NTR^/TrkA ratio [38]. Thus, our data suggest that the NGF signaling deregulation in the skin may induce the DRG atrophy and degeneration. The latter has indeed been reported in diabetic states [18, 39] as a consequence of an axonal retraction and cellular secondary death reaction [40, 41]. In the spinal cord, a prevalent increase in tissue and terminals intensity for TrkA receptor in diabetic rats (Table 3) has been found. TrkA is involved in NGF retrograde transport; thus, our data suggest that the NGF increased levels found in the DRG may be of spinal origin [32].

EA could revert neuropathic symptoms in the early diabetes [3], and its action is possibly exerted on mediators of DRGs neuron sensitization, such as NGF, MAPKs, and the TRPV1. Western perspective assumes that a potent sensory stimulation is elicited by needle insertion and stimulation, resulting in local, spinal/segmental, and central modulation of neuronal activity [15, 17]. Acupuncture-based techniques exert their therapeutic action regulating the activity-dependent production and release of neuromodulators, neuropeptides and neurotrophins [16, 42, 43]. The analgesic effects of EA have been correlated to a decreased activity of the p38 signaling pathway in the spinal cord [44] and to an increased Nf-*κ*B activity [45]. Our results indicate that EA influences NGF protein levels rather than the NGF gene expression. The actions of EA on DPN could descend from a possible activity-dependent modulation of the NGF system activity, with concomitant actions on spinal GABAergic and/or opioidergic neurotransmission [3, 46–49], rather than on possible general improvement of glucose metabolism, as indicated by the observed lack of effects of EA on plasma glucose and insulin. The normalization of NGF signaling by EA in early diabetes could also be of neuroprotective relevance to DRG neurons. Indeed, our overall results suggest an EA-driven decrease of p38 and JNK proapoptotic signaling [38] and a possible reduction of neuronal hyperexcitability driven by the TRPV1 channel [8, 30].

The anatomical location for EA-induced NGF modulation may be the spinal cord. We previously demonstrated that early STZ-induced thermal hyperalgesia could be reverted by EA and that this effect was parallel with a decrease in NGF and TRPV1 content and TrkA activation, all found in the spinal cord rather than in the paw skin [3]. Our present report demonstrates that STZ modulates NGF/NGF receptors and also TRPV1 in the DRGs. The EA-induced modulation of NGF signaling in the DRG spinal circuitry represents a novel possible mechanism by which EA could normalize sensory perception in neuropathic states by controlling the neuronal sensitization stimulated by hyperneurotrophism. EA effects on the spinal supply of NGF to DRG could be of importance in our experimental model, while the accumulation of NGF previously found in the peripheral field [3] could reflect a decreased transport of the neurotrophin from the skin to sensory neurons, secondary to the diabetes-induced peripheral functional denervation [40, 41]. Interestingly, we found that EA was able to partially revert the STZ-induced p75^NTR^ upregulation in the skin, suggesting a selective contrast to NGF deregulation. Possible mechanisms that remain to be specifically addressed in this latter EA action encompass EA-regulated antidromic modulation of sensory activity [50] and/or sympathetic drive [51] to the skin, both able to regulate NGF by challenging neuropeptides and adrenergic receptors, respectively [52]. Of note, EA was able to normalize spinal TrkA levels, as resulting from confocal microscopy (Table 3), and most probably NGF transport/accumulation in the spinal DRG circuit. However, while for the skin we dissected the basal cell layer from the DRG contribution, in the spinal cord we could not dissect the central and DRG contributions to NGF-receptors expression, but this is an important point which needs further investigations.

The previous literature, both on animal models and in humans, shows that late DPN is characterized by a defective NGF support to sensory neurons, due to reduced NGF production and/or transport from target tissues [10]. Supported by this rationale, several clinical trials were attempted based on the administration of NGF in human DPN [7, 10, 11]. The overall results of such trials indicated that the benefits of NGF treatment were overcome by the occurrence of hyperalgesia and pain, as side effects linked to the high dosage of NGF needed to achieve a positive therapeutic outcome [7]. In our present and previous [3] works, we investigated, for the first time, the relevance of NGF in the early establishment of diabetic neuropathy. According to our data and to the theory of DPN as a biphasic process, characterized by two distinct developmental stages [1], the NGF could assume a double role: in the first time of diabetes development it could actively participate in the establishment of neuronal sufferance and of the hyperalgesic symptoms; in a late phase it could be of therapeutic value, being able to support suffering DRG neurons and reestablish impaired sensory functions. In both cases, a therapy with EA could be valuable in the care of DPN. Indeed, being the development of DPN at an early stage, EA could counteract the DPN-associated hyperneurotrophism and exert a neuroprotective action on peripheral neurons. On the other hand, in a late stage, EA could be of a supportive value if associated with NGF therapy, being able to counteract NGF-induced hyperalgesic symptoms [7, 53].

In conclusion, NGF-triggered DRG neuron hyperactivity could be of primary importance in the establishment of DPN and in the early diabetes-associated neuronal distress. Physical therapies based on sensory stimulation, such as EA, could be valid supportive tools in controlling DPN development and progression and hopefully for the neuro-protection of diabetes-targeted DRG neurons.

## Supplementary Material

Supplemental Figure 1: Low magnification confocal images of TrkA and p75^NTR^ double immunofluorescence in the epidermis/dermis in STZ, STZ+EA and Control groups.
In all three groups, TrkA immunoreactivity appeared mainly confined to the epidermis, while p75^NTR^ was distributed to the both epidermal and dermal regions. However, many of the sensory, glandular and muscular structures present in the dermis expressed a high intensity p75^NTR^ immunofluorescence and a medium intensity TrkA immunofluorescence. TrkA in the epidermis was densely distributed in the granular-squamous and basal epidermal layers as a medium tissue background decorated by small vesicles endowed of medium to high intensity fluorescence. On this tissue background, and selectively confined to the proliferating basal cell layer, small vesicles of high intensity fluorescence densely filled the cellular cytoplasm giving more evidence to the basal cell layer. However, very often, in this latter layer, rows of cells which did not show the intense TrkA immunofluorescence were alternated to rows of cells showing the intense TrkA immunofluorescence. p75^NTR^ immunoreactivity was present as low-medium intensity tissue background in the epidermal region with the exception of few positive vesicles located in the basal cell layer. Of interest, in the dermal papillae and in juxtaposition to the epidermal basal cell layer, several sensorial and glandular structures often showed an intense p75^NTR^ immunoreactivity.

## Figures and Tables

**Table 1:** Summary of antibodies used for western blot analysis.

Antibody	Species	Clone/cat.	Supplier	Dilution
TrkA	Rabbit	118	Santa Cruz	1 : 1000
p_Tyr496_-TrkA	Rabbit	8058	Santa Cruz	1 : 1000
p75^NTR^	Mouse	Clone 192	Intramural	1 : 2000
ERK1/2	Rabbit	4067	Cell signaling	1 : 1000
p_Thr218-Tyr220_-ERK1/2	Rabbit	3371	Cell signaling	1 : 1000
AKT	Rabbit	4685/11E7	Cell signaling	1 : 1000
p_Thr308_-AKT	Rabbit	4056	Cell signaling	1 : 1000
JNK	Rabbit	3708	Cell signaling	1 : 1000
p_Thr183-Tyr185_-JNK	Rabbit	4668	Cell signaling	1 : 1000
p38	Rabbit	9212	Cell signaling	1 : 1000
p_Thr180-Tyr182_-p38	Rabbit	4631	Cell signaling	1 : 1000
Phospho-NF-*κ*B-p65	Rabbit	6956	Cell signaling	1 : 1000
Phospho-I-*κ*B-*α*	Rabbit	5209	Cell signaling	1 : 1000
TRPV-1	Rabbit	28759	Santa Cruz	1 : 1000
GAPDH	Rabbit	25778	Santa Cruz	1 : 5000

**Table 2:** Analysis of morphometric and colocalization indexes for the three experimental groups (CTRL, STZ, and STZ + EA) after a mask was applied to isolate the basal cell layer of the skin and laminae I and II in the spinal cord.

Morphometry						Colocalization		
Skin		Tissue intensity	Vesicles number	Vesicles intensity	Diameter (*μ*m)	Pearson's coefficient	Manders' coefficient TrkA/p75^NTR^	Manders' coefficient p75^NTR^ /TrkA
CTRL	TrkA	33.59 ± 1.40	594 ± 71.52	151.83 ± 26.51	0.56 ± 0.09	0.18 ± 0.06	0.02 ± 0.01	0.79 ± 0.08
	p75^NTR^	5.55 ± 2.78	63 ± 25.48	15.27 ± 8.29	0.38 ± 0.03			
STZ	TrkA	44.26 ± 6.81	643 ± 119.18	111.80 ± 21.05	0.44 ± 0.08	0.01 ± 0.04	0.11 ± 0.01	0.64 ± 0.05
	p75^NTR^	14.27 ± 0.34	193 ± 50.50	75.03 ± 24.74	0.46 ± 0.04			
STZ + EA	TrkA	44.93 ± 6.43	511 ± 93.38	115.96 ± 22.65	0.50 ± 0.13	0.01 ± 0.03	0.15 ± 0.12	0.63 ± 0.08
	p75^NTR^	12.12 ± 2.03	83 ± 13.00	38.13 ± 18.35	0.34 ± 0.18			

Spinal cord		Tissue intensity	Terminals number	Terminals intensity	Diameter (*μ*m)			

CTRL	TrkA	30.62 ± 8.52	634 ± 31.18	97.53 ± 9.50	2.21 ± 0.42	0.05 ± 0.05	0.63 ± 0.26	0.36 ± 0.17
	p75^NTR^	37.66 ± 11.82	886 ± 48.74	87.52 ± 5.91	1.42 ± 0.55			
STZ	TrkA	47.73 ± 5.68	677 ± 21.65	127.83 ± 10.67	1.79 ± 0.18	0.06 ± 0.07	0.19 ± 0.18	0.57 ± 0.12
	p75^NTR^	26.00 ± 3.26	687 ± 150.19	49.51 ± 5.70	2.18 ± 0.39			
STZ + EA	TrkA	23.91 ± 3.5	676 ± 52.88	69.84 ± 19.90	1.66 ± 0.17	0.01 ± 0.01	0.34 ± 0.09	0.21 ± 0.08
	p75^NTR^	29.18 ± 3.13	725 ± 89.55	71.52 ± 23.52	1.77 ± 0.31			

Morphometry describes the total fluorescence intensity of the tissue isolated in the mask, the vesicles or terminal numbers, their fluorescence intensity, and their diameter. Data are reported as mean ± SD and obtained by one-way ANOVA. Tukey's test and variation analysis performed on data presented in Table 2 are reported in Table 3.

Colocalization Pearson's coefficient describes the relationship between the pixel intensities of the two channels by linear regression. Values −1 to 0.5 indicate absence of colocalization and values 0.5 to 1 indicate colocalization. Manders' coefficient indicates the proportion of signal of one channel overlapping with signals of another channel. Values <0.5 indicate absence of overlapping; values >0.5 indicate overlapping between channels.

**Table 3:** Post hoc comparisons within different sets of data presented in Table 2 performed using the Tukey's HSD test.

Skin		Tissue intensity	Vesicles number	Vesicles intensity	Diameter (*μ*m)
STZ/CTRL	TrkA	↑NS	↑NS	↓NS	↓NS
	p75^NTR^	↑*	↑*	↑*	↑NS
STZ + EA/STZ	TrkA	=	↓NS	=	↑NS
	p75N^TR^	↓NS	↓*	↓NS	↓NS

Spinal cord		Tissue intensity	Terminals number	Terminals intensity	Diameter (*μ*m)

STZ/CTRL	TrkA	↑*	↑NS	↑*	↓NS
	p75^NTR^	↓NS	↓NS	↓*	↑NS
STZ + EA/STZ	TrkA	↓*	=	↓NS	↓NS
	p75^NTR^	↑NS	↑NS	↑NS	↓NS

↑: increase. ↓: decrease. =: no variation. NS: not significant; *P < 0.05.

## Abbreviations

DRG: Dorsal root ganglion
EA: Electroacupuncture
p-JNK: Phospho-c-jun N-terminal kinase
Nf-*κ*B: Nuclear factor kappa light chain enhancer of activated B cells
NGF: Nerve growth factor
p-p38: Phospho-p38
p75^NTR^: p75 neurotrophin receptor
STZ: Streptozotocin
p-TrkA: Phospho-tyrosine kinase A receptor
TRPV1: Transient receptor.
