# Supplementary material for: Increased Nerve Growth Factor Signaling in Sensory Neurons of Early Diabetic Rats Is Corrected by Electroacupuncture
Source: Evid Based Complement Alternat Med. 2013 Apr 21;2013:652735. doi: 10.1155/2013/652735 (PMC3654322; doi:10.1155/2013/652735)
Supplement: Supplementary file 1 — Supplemental Figure 1: Low magnification confocal images of TrkA and p75NTR double immunofluorescence in the epidermis/dermis in STZ, STZ+EA and Control groups. In all three groups, TrkA immunoreactivity appeared mainly confined to the epidermis, while p75NTR was distributed to the both epidermal and dermal regions. However, many of the sensory, glandular and muscular structures present in the dermis expressed a high intensity p75NTR immunofluorescence and a medium intensity TrkA immunofluorescence. TrkA in the epidermis was densely distributed in the granular-squamous and basal epidermal layers as a medium tissue background decorated by small vesicles endowed of medium to high intensity fluorescence. On this tissue background, and selectively confined to the proliferating basal cell layer, small vesicles of high intensity fluorescence densely filled the cellular cytoplasm giving more evidence to the basal cell layer. However, very often, in this latter layer, rows of cells which did not show the intense TrkA immunofluorescence were alternated to rows of cells showing the intense TrkA immunofluorescence. p75NTR immunoreactivity was present as low-medium intensity tissue background in the epidermal region with the exception of few positive vesicles located in the basal cell layer. Of interest, in the dermal papillae and in juxtaposition to the epidermal basal cell layer, several sensorial and glandular structures often showed an intense p75NTR immunoreactivity. [file 652735.f1.docx]

**Supplemental Material**

# Supplemental Figure 1

*Low magnification confocal images of TrkA and p75^NTR^ double immunofluorescence in the epidermis/dermis in STZ, STZ+EA and Control groups.*

In all three groups, TrkA immunoreactivity appeared mainly confined to the epidermis, while p75^NTR^ was distributed to the both epidermal and dermal regions. However, many of the sensory, glandular and muscular structures present in the dermis expressed a high intensity p75^NTR^ immunofluorescence and a medium intensity TrkA immunofluorescence. TrkA in the epidermis was densely distributed in the granular-squamous and basal epidermal layers as a medium tissue background decorated by small vesicles endowed of medium to high intensity fluorescence. On this tissue background, and selectively confined to the proliferating basal cell layer, small vesicles of high intensity fluorescence densely filled the cellular cytoplasm giving more evidence to the basal cell layer. However, very often, in this latter layer, rows of cells which did not show the intense TrkA immunofluorescence were alternated to rows of cells showing the intense TrkA immunofluorescence. p75^NTR^ immunoreactivity was present as low-medium intensity tissue background in the epidermal region with the exception of few positive vesicles located in the basal cell layer. Of interest, in the dermal papillae and in juxtaposition to the epidermal basal cell layer, several sensorial and glandular structures often showed an intense p75^NTR^ immunoreactivity.

**Supplemental Figure 1**

**
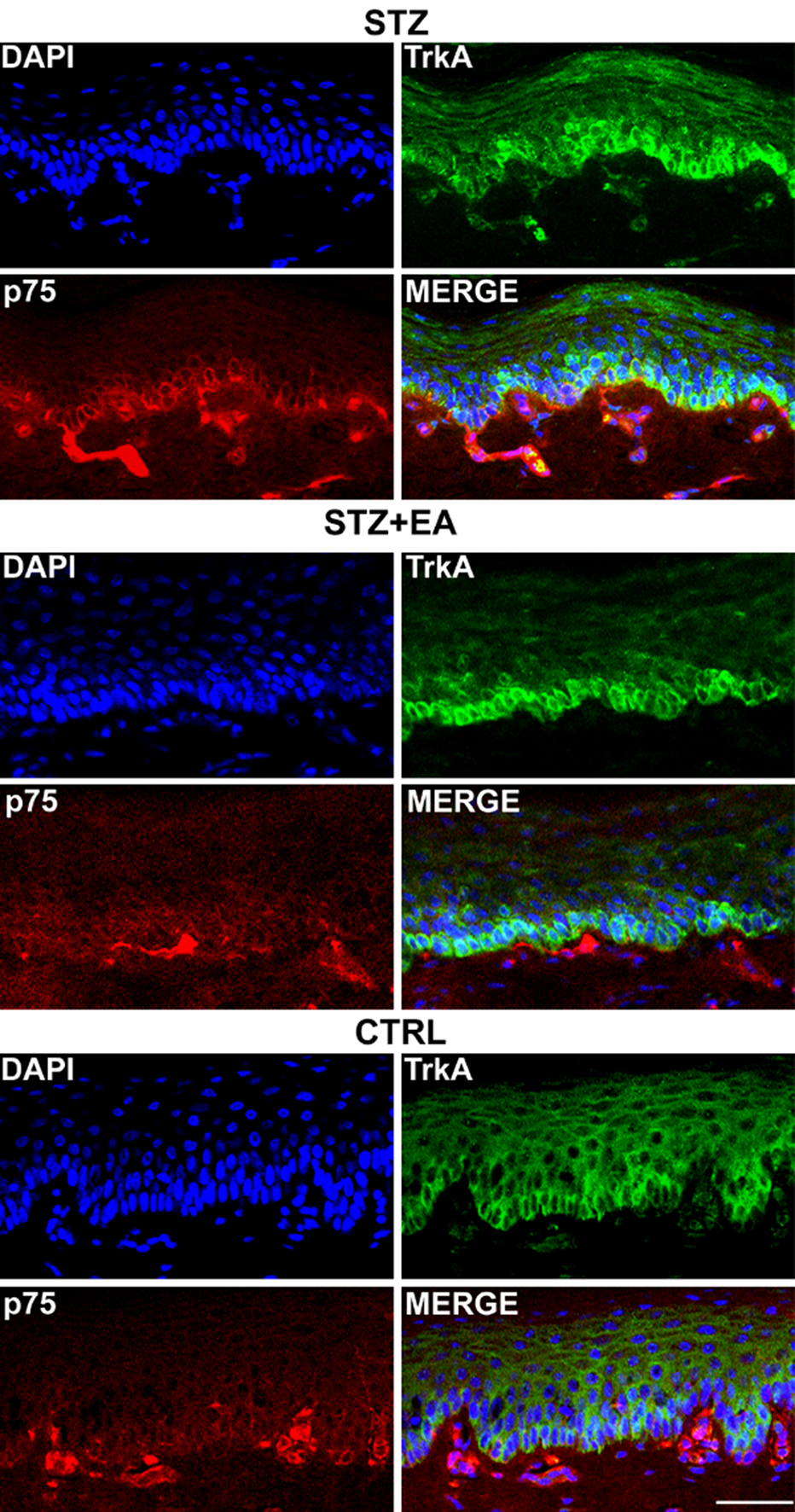
**
